# Milestones in *Vibrio* Science and their Contributions to Microbiology and Global Health

**DOI:** 10.5334/aogh.4711

**Published:** 2025-05-13

**Authors:** Lapo Doni, Elisa Taviani, Emanuele Bosi, Carla Pruzzo, Jaime Martinez-Urtaza, Luigi Vezzulli

**Affiliations:** 1 Department of Earth, Environmental and Life Sciences (DISTAV), University of Genoa, Genoa, Italy; 2 NBFC, National Biodiversity Future Center, Palermo, Italy; 3 Department of Genetics and Microbiology, Universitat Autònoma de Barcelona (UAB), Barcelona, Spain

**Keywords:** Pathogenic bacteria, History, Cholera, Public health

## Abstract

*Background: Vibrio*, a group of Gram‑negative bacteria found in the ocean, has become a significant global threat, intensified by climate change, owing to its crucial roles in environmental, human, and animal health. Research on these bacteria and the diseases they cause has greatly influenced scientific progress, resulting in major advancements in the fields of microbiology, epidemiology, and public health.

*Objectives:* This review aims to highlight the early groundbreaking discoveries in *Vibrio* research, particularly those that have significantly impacted the science of microbiology and global health.

*Methods:* A comprehensive literature search was conducted across vast databases of biomedical and life sciences literature including PubMed, EMBASE, and Scopus. Additionally, a search of the grey literature was performed. Studies that marked early groundbreaking discoveries in *Vibrio* research, with wide implications for human society, were included.

*Findings and conclusion:* Research on *Vibrio* has led to major advancements in our understanding of disease mechanisms, pathogen ecology, and the epidemiology of waterborne infections. A landmark discovery was the identification of *Vibrio cholerae* in 1884, which played a crucial role in studying waterborne diseases such as cholera and led to the development of modern approaches to treat diarrheal diseases, such as the introduction of oral rehydration salt (ORS) therapy. Certain *Vibrio* strains, such as *Vibrio vulnificus*, are important models for studying flesh‑eating diseases, while others, such as *Vibrio parahaemolyticus* ST3, ST36, and *V. cholerae* O1, are the only marine bacteria known to cause global epidemics by spreading across continents. Key mechanisms in Gram‑negative bacteria, including the viable but nonculturable (VBNC) state, quorum sensing, and the type VI secretion system (T6SS), were first discovered in *Vibrio* species. Today, research on *Vibrio* bacteria remains crucial from a global health perspective, especially owing to the expanding effects of climate change on their worldwide distribution.

## Introduction

**Figure 1:**
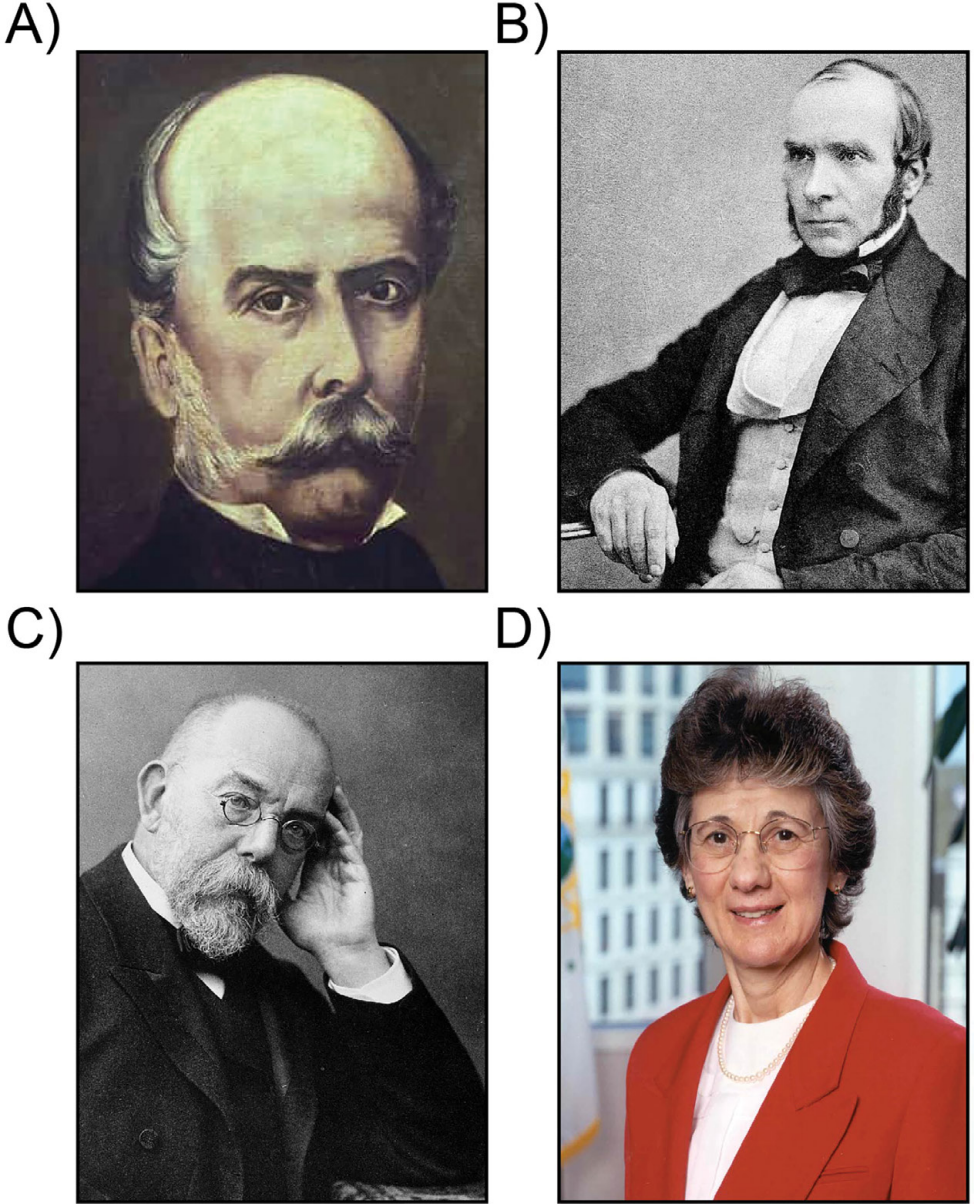
In the nineteenth century, Filippo Pacini **(a)**, John Snow **(b)**, and Robert Koch **(c)** left an indelible mark on the history of cholera and are rightly considered the “fathers” of studies on the disease and its causative microorganism (see text for details). In modern times, Rita R. Colwell **(d)** stands out as one of the most influential microbiologists in cutting‑edge research on *Vibrio*. In particular, she is promoting understanding of the biology, ecology, and impact of these bacteria on global public health.

Throughout history, infectious diseases have devastated human populations, causing widespread fear. This is certainly true for *Vibrio* pandemics, which have substantially impacted human society. For example, cholera outbreaks terrified people because of their sudden onset, rapid spread, and horrific symptoms, including massive rice‑water diarrhea, bleeding fluid from the mouth, and violent muscle contractions that continued even after death, occurring within hours or days. However, the *Vibrio* genus does not only include pathogens but also species with complex mutualistic relationships, with wide range of metabolic capabilities, and also inhabitants of the deep ocean [1]. The term “*Vibrio*” is derived from the Latin word “*vibrare*,” which means to vibrate or move rapidly, and it is considered one of the earliest names for a bacterial taxon [2]. The common ancestor of all *Vibrio* species is estimated to have existed around 600 million years ago [3]. During this long period, the lineage has undergone profound genetic changes and adaptations, allowing its members to evolve in a variety of habitats, from marine and freshwater ecosystems to the guts of animals and even plants [4]. Historically, much of the research and literature on *Vibrio* originated in the field of medical microbiology, focusing primarily on *Vibrio cholerae*, the causative agent of cholera. In contrast, research on *Vibrio* species commonly found in aquatic ecosystems has been conducted by pathologists and environmental microbiologists, particularly those exploring pathogenic bacteria causing infections in marine animal hosts [5]. In both cases, many prestigious and influential microbiologists have contributed significantly to *Vibrio* research (Figure 1), and the study of *Vibrio* species has led to important achievements in microbiology, such as the discovery of the viable but nonculturable (VBNC) physiological state [6], the quorum sensing cellular signaling [7], and the type VI secretion system (T6SS) in Gram‑negative bacteria [8].

The rationale for this review is not to comprehensively cover every relevant aspect of *Vibrio* science, but to specifically emphasize early breakthrough discoveries in *Vibrio* research, one of the most enigmatic bacterial genera, particularly those that have had a profound impact on the science of microbiology and on human and animal life [9–11]. To this end, the review is structured in two parts, chronologically following the major *Vibrio* discoveries over the years: the history of *V*. *cholerae* and the history of non‑cholera *Vibrio* species.

## The History of *Vibrio* Genus: Cholera and *Vibrio* Cholerae (Figure 2)

**Figure 2:**
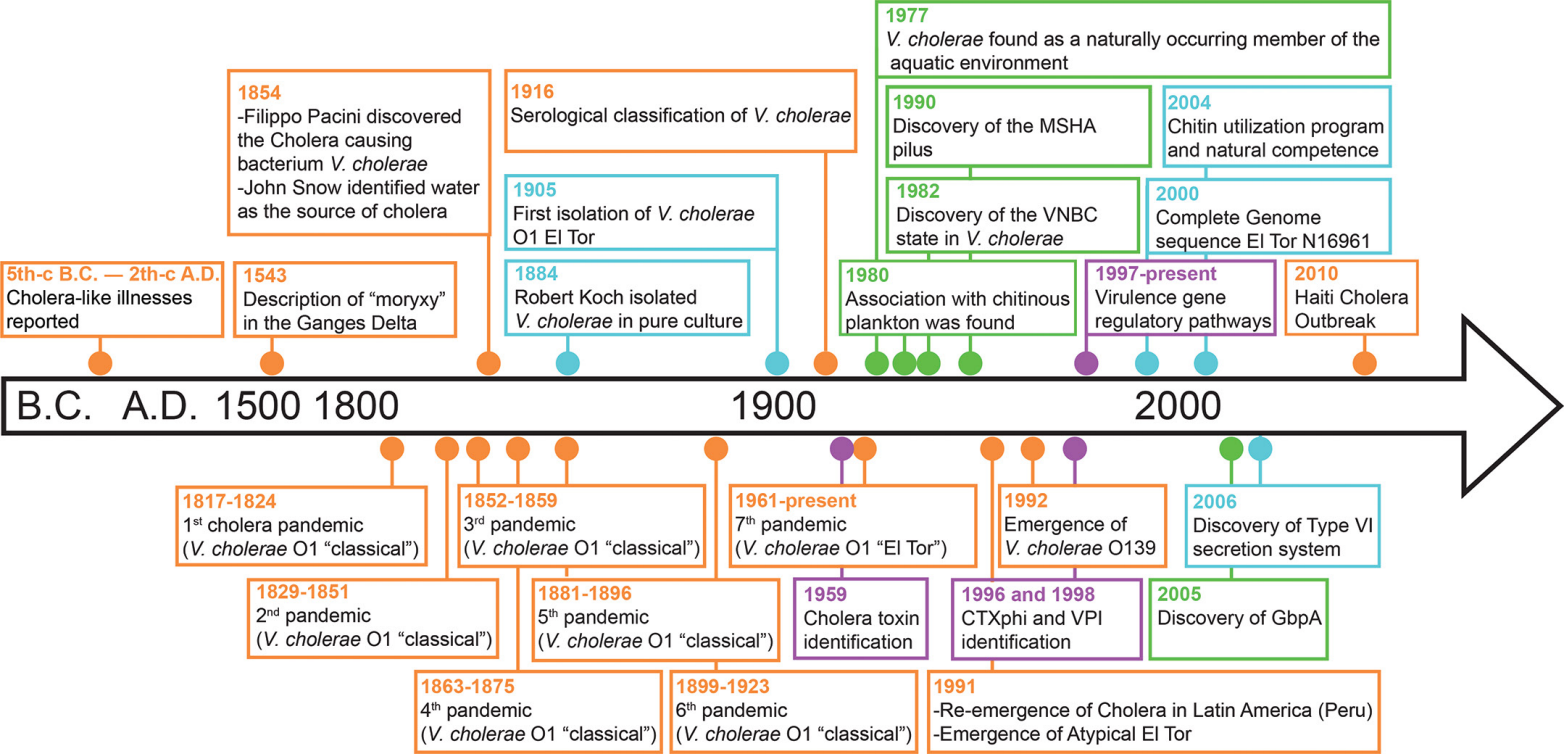
Overview and timeline of historically notable discoveries regarding cholera and *Vibrio cholerae*. Key moments are indicated by dots color‑coded on the basis of their focus: epidemiology (**orange**), biology and evolution (**blue**), virulence (**purple**), and ecology (**green**).

Cholera, caused by the bacterium *V*. *cholerae*, has been a major human scourge for centuries. Today, *Vibrio cholerae* infects an estimated 1.3 to 4 million people annually, resulting in 21,000 to 143,000 deaths, and has historically posed a health threat in at least 175 countries worldwide [12]. *V. cholerae* is classified according to the O‑antigen polysaccharide properties of its outer membrane, with serogroups O1 and O139 being the primary strains responsible for causing cholera pandemics and epidemics. In contrast, non‑O1/non‑O139 strains (belonging to non‑cholera vibrios) typically cause milder forms of gastrointestinal infections. The etymology of the term “cholera” has been debated for many years, but it may provide hints to understanding the disease [9]. The word “cholera” may originate from the Greek words, “*chole*” (bile) and “*rein*” (to flow), meaning the flow of bile [13]. Other researchers suggest that it derives from the Greek word “*cholera*,” meaning gutter [14]. The symptoms of cholera may have reminded the Greeks of the rapid flow of water through gutters during a downpour [13].

### Up to the third century

The earliest known records of cholera‑like illnesses can be traced to ancient Sanskrit writings in India in the fifth century BC and later in the writings of Hippocrates (460–377 BC), Galen (129–216 AD), Aretaeus of Cappadocia (first and second century AD), and Wang Shuhe (180–270 AD) [15].

Several pieces of ancient Indian evidence also described a disease similar to cholera [16]. For instance, there was in a temple at Gujrat in western India, a monolith dating back to the time of Alexander the Great, with the following inscription, referring apparently to cholera:

“The lips blue, the face haggard, the eyes hollow, the stomach sunk in, the limbs contracted and crumpled as if by fire, those are the signs of the great illness which, invoked by a malediction of the priests, comes down to slay the braves ... ”

However, there is no evidence that these early records referred specifically to infections caused by *V. cholerae*, nor is it clear whether the disease manifested in the same epidemic way as is known today [11].

### Fifteenth to seventeenth century

The erliest documented cases of cholera date back to the fifteenth century in India, following the arrival of Portuguese explorer Vasco da Gama in 1498. In 1503, an epidemic of cholera was reported in the army of Calicut, where about 20,000 men died from a disease that struck them suddenly in the abdomen, with some dying within 8 h. This was followed by another outbreak in the population in 1543. The Portuguese historian Gaspar Correa, author of *Legendary India*, gave one of the first descriptions of the clinical conditions of a “cholera‑like” epidemic called “*moryxy*” in the Ganges Delta in 1543. The first documented reference to a cholera outbreak outside of India dates to the year 1629 and occurred in Jakarta [15].

### Nineteenth century

Despite the disease having been likely present since the dawn of humanity, the first documented pandemic of cholera emerged in 1817 in the Ganges Delta in India, most probably originating from contaminated rice. The disease quickly spread throughout most of India and by 1822 reached Thailand, Indonesia, the Philippines, China, and Japan, killing thousands of people. Before ending in 1824, the pandemic had spread beyond Asia, carried by British troops traveling from India to Oman and reaching the Persian Gulf and Europe (Turkey, Syria, and southern Russia). After the first pandemic, five more cholera pandemics caused by *V. cholerae* classical biotype O1 were reported in the nineteenth century (1829, 1852, 1863, 1881, and 1899), all emerged from Bengal region.

The significant turning point in the history of the genus *Vibrio* occurred during the third cholera pandemic, widely recognized as the deadliest in history. Once again originating in India, this pandemic caused a large number of victims across Asia, Europe, Africa, and North America, with 1854 being a particularly devastating year. In the same year, John Snow became famous for tracing a cholera outbreak to a single contaminated well [17]. This discovery was a milestone in the field of epidemiology and is still considered a canonical example today. Snow’s work demonstrated that contaminated water was the source of the outbreak by tracing back the houses of cholera patients living in the Soho district of London, leading him to conclude that contaminated water from the Broad Street pump was the source of the disease. As a result, the removal of the pump handle led to the end of the epidemic. Snow correctly identified the fecal–oral route as the transmission mode of human infection [17], contributing significantly to public health. In recognition of his pioneering studies, the John Snow Society promotes the scientific legacy of Snow was founded in 1992 in London with the support of the London School of Hygiene and Tropical Medicine and the Royal Society for Public Health. However, despite his efforts, Snow failed to identify the specific pathogen responsible for causing cholera. Thus, the predominant belief that cholera was an airborne disease persisted [18].

The same year, Filippo Pacini, a researcher in Florence, discovered that a germ was the cause of cholera. Utilizing microscopy during autopsies of cholera victims, Pacini observed a close association between the rupture of the intestinal lining and millions of bacteria, which he named *Vibrio cholerae*. Using his histological techniques, Pacini determined that the intestinal mucosa dysfunction led to debilitating symptoms such as diarrhea, vomiting, severe dehydration, and death [19]. Unfortunately, his discovery was not recognized during his lifetime. Indeed, this did not happen until 82 years after his death, when the international committee on nomenclature in 1965 adopted *Vibrio cholerae* Pacini 1854 as the correct name of the cholera‑causing organism [20]. During the same year and around the same period [11], toward the end of August, while in Barcelona there was a peak in cholera cases, Spanish pharmacist Joaquín Balcells y Pascual placed an open glass of pure water near a cholera patient, exposing it to the patient’s exhalations for 3 days. Although the water remained visually clear, a whitish deposit formed at the bottom of the glass. Using his microscope, Balcells observed numerous vibrios exhibiting remarkable mobility, characterized by angular movements [21]. Simultaneously, in Great Britain, the Report of the Committee for Scientific Inquiries in relation to the cholera epidemic of 1854 was being compiled by the General Board of Health of the Medical Council and the Committee for Scientific Inquiries. The report claimed the presence of vibrios in the air of the cholera ward, which was filled with patients. However, the report concluded that it was premature to infer a connection between the disease and these organisms just because they were widespread [22], regardless of the fact that cholera airborne transmission has never been able to be proven.

However, it took nearly 30 years to achieve the first isolation of the causative agent of cholera in pure culture [11]. On 7 January 1884, Robert Koch announced that he had successfully isolated the bacillus in pure culture from cholera‑affected patients in Calcutta (India). Koch provided a detailed description of the organism, improving the understanding of *V. cholerae* and its role in causing cholera [23]. This discovery had a huge impact on the understanding of infectious diseases and public health, leading to the development of specific preventions and treatments for cholera. Indeed, the following year, in 1885, the Catalan bacteriologist Jaime Ferran y Clua formulated for the first time the cholera vaccine, based on live bacteria [24].

While the first six cholera pandemics were caused by strains of the O1 classical biotype, in 1905, a new strain of *V. cholerae* serogroup O1, today referred to as biotype El Tor, was isolated from pilgrims in a quarantine camp located in the City of El Tor in Egypt. El Tor strains were associated with sporadic cholera and localized epidemics before emerging as the causative agent responsible for the seventh pandemic in 1961.

### Twentieth and twenty‑first centuries

Unlike earlier pandemics, which originated in the Ganges Delta region the seventh and current cholera pandemic caused by the *V. cholerae O1* El Tor biotype began on the Island of Celebes (Sulawesi) in Indonesia in 1961, spreading initially across Asia and reaching Africa in 1971, the continent currently most affected by or reporting most cholera cases in the world according to the World Health Organization. Unexpectedly, in January 1991, cholera reemerged simultaneously in many coastal cities of Peru, after over 100 years of absence from South America, killing more than 3,000 people in the region before spreading to other countries in South and Central America [25].

In 1992, a new serogroup, namely *V. cholerae* serogroup O139, with epidemic potential, arose in India and Bangladesh, prompting concerns that this could represent the beginning of the eighth pandemic. Around the same time, between 1991 to 1994, ‘atypical El Tor’ variants with attributes of the classical biotype were isolated from hospitalized patients with acute diarrhea in Matlab, Bangladesh [26] and they later became the dominant cause of cholera globally [27].

More recently, the 2010 cholera epidemic in Haiti stands out as one of the most significant public health crises in recent history, in terms of both its devastating scale with over 10,000 deaths and hundreds of thousands of infections and its prolonged duration [28]. The outbreak struck a country already reeling from the catastrophic earthquake of January 2010, compounding the humanitarian crisis and overwhelming an already fragile healthcare infrastructure. Beyond its national impact, the epidemic had profound international implications. It marked the first time in history that the United Nations, having been implicated in the introduction of the disease through peacekeeping forces, issued an official apology to a member state. This event sparked global discussions about accountability, international health governance, and the ethical responsibilities of humanitarian organizations in crisis zones.

The twentieth and twenty‑first centuries have also witnessed major advancements in the understanding of the biology and pathogenicity of *V. cholerae*, which are far beyond the scope of this paper and have been thoroughly reported elsewhere [29]. Initial “fingerprinting” of *V. cholerae* strains began in 1916, leading to serological classification [30]. In 1959, Sambhu Nath De discovered the cholera toxin (CT) by observing that cell‑free *V. cholerae* culture filtrate was capable of eliciting massive accumulation of “rice‑water” fluid in the ligated ileal loops of adult rabbits [31]. CT was later found to be encoded by a filamentous phage, CTXΦ, which can integrate into the host *V. cholerae* genome, fostering lysogenic conversion [32]. Discoveries of CTXΦ and *Vibrio* pathogenicity islands (VPIs) encoding major virulence factors (e.g., CT and co‑regulated pilus‑TCP) marked significant turning points in cholera science. CT, which is encoded by *V. cholerae* O1 and O139 and is rarely found in other non‑epidemic serotypes, was further purified and characterized, revealing that it consists of two subunits, A and B [1, 33]. The B subunit binds the GM1 ganglioside eukaryotic receptor on human intestinal cell membranes, triggering toxin A internalization via endocytic vesicles, which in turn foster watery diarrhea by a transepithelial osmotic gradient that causes water flow into the lumen of the intestine [1].

Following the discovery of CT, key pathways regulating virulence genes such as multiple signal transduction circuits were uncovered [29, 34]. In 2006, the type VI secretion system (T6SS), used by a wide range of Gram-negative bacteria to transport effectors from the interior of a bacterial cell across the membrane into an adjacent target cell, was first discovered in *V. cholerae* by John Mekalanos and colleagues at Harvard Medical School in Boston [8]. From a public health perspective, unraveling the pathogenesis mechanisms causing cholera had important repercussions for the treatment of diarrheal diseases.

Oral rehydration therapy was developed in the 1960s following the discovery that the glucose‑mediated cotransport of sodium and water across the mucosal surface of the small intestine epithelium continues to function during cholera infection, even in the presence of cholera toxin. In 1964, Captain Phillips of the US Army was the first to successfully administer oral glucose saline to cholera patients. Building on this, researchers at the Cholera Research Laboratory in Dhaka and the Infectious Diseases Hospital in Calcutta played key roles in the development of the modern oral rehydration salt (ORS) solution.

Starting from the second half of the nineteenth century, major scientific breakthroughs were also made in the field of *V. cholerae* ecology. Notably, more than a century after John Snow discovered a link between contaminated water and cholera disease, the *V. cholerae* bacterium was finally proved to be an indigenous microorganism of brackish and coastal marine waters [35]. Subsequently, studies by Rita Colwell and collaborators have shown that *V. cholerae* persists in the marine environment mainly in association with zooplankton organisms, which represent the bacterium’s most important environmental reservoir [36]. This discovery also had a profound impact from a sanitation perspective, as it was found that simple and accessible water filtration methods using domestic sari cloth in Bangladeshi villages provided a cost‑effective and immediate solution to remove plankton‑associated *Vibrio* strains and reduce cholera transmission in areas with limited access to advanced water purification technologies [37]. Colonization factors such as chitin binding proteins and the mannose‑sensitive hemagglutinin (MSHA) pilus mediate *V. cholerae* O1 attachment to the exoskeleton of plankton organisms [38]. Among these, an outer membrane protein named *N*‑acetyl glucosamine (GlcNAc)‑binding protein A (GbpA) was found to be involved in *V. cholerae* classical O1 attachment to chitin and adhesion to cultured intestinal epithelial cells, providing a direct link between *V. cholerae* survival strategies in the aquatic environment and human infection [39, 40]. *V. cholerae* was found to possess a molecular program for the efficient utilization of chitin and the ability to acquire exogenous genetic material by natural transformation during growth on this substrate [41, 42]. It is now well known that chitin has a profound influence on *V. cholerae* ecology [43].

Simultaneously with the discovery of *V. cholerae* being a marine bacterium, the viable but nonculturable (VBNC) state was revealed, adding another layer to the complexity of understanding of this pathogen [6]. VBNC is a unique survival strategy of bacteria in the environment in response to harsh environmental conditions. VBNC bacteria cannot be cultured on traditional microbiological media, but they remain viable, and once the environmental conditions become favorable again, they resuscitate and can even become virulent. The VBNC phenomenon may explain the sporadic nature of cholera outbreaks and the inability to recover *V. cholerae* from natural waters between epidemics [11]. These findings had substantial implications for understanding the pathogenesis of this bacterium and for the study of microorganisms’ physiology. In the early 2000s, the explosion in genomics and sequencing techniques, as well as the implementation of advanced microscopic, biochemical, and molecular approaches, enabled additional significant breakthroughs in cholera research [9]. Notably, the complete genome sequence of *V. cholerae* El Tor N16961, released in 2000, provided insights into the environmental and pathobiological characteristics of this microorganism. A relevant finding was that *V. cholerae* possessed two chromosomes, a genomic peculiarity, which has been later showed to be shared across the *Vibrio* genus [44].

After nearly 200 years of studying cholera and its causative bacterium, *V*. *cholerae* remains a model organism in bacteriology and the biological sciences.

## The History of *Vibrio* Genus: Non‑Cholera *Vibrio* Species (Figure 3)

**Figure 3:**
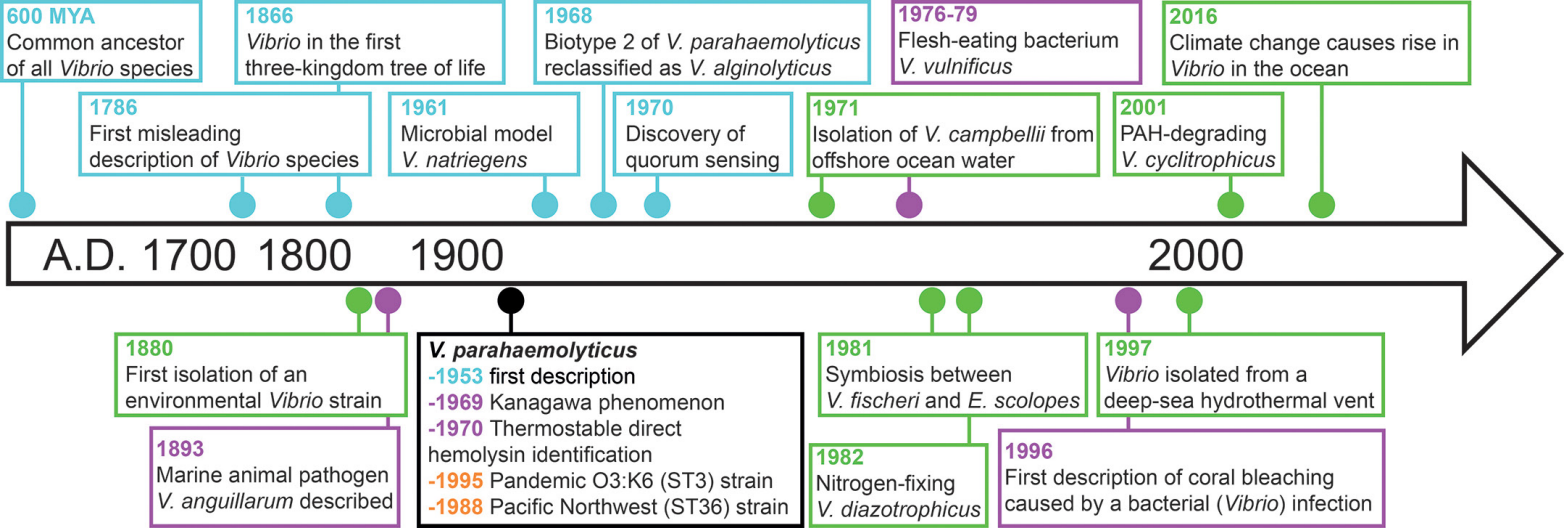
Overview and timeline of historically notable discoveries in non‑cholera *Vibrio* species. Key moments are color‑coded dots on the basis of their focus: epidemiology (**orange**), biology and evolution (**blue**), virulence (**purple**), and ecology (**green**).

Non‑cholera *Vibrio* species are environmental organisms found in marine and estuarine waters. While they do not cause cholera, many can still be pathogenic to humans and animals, particularly through exposure to water or seafood where these bacteria are present. Others have evolved complex symbiotic relationships with their hosts or play essential roles in aquatic ecosystems. Notably, non‑cholera *Vibrio* infections linked to exposure through water or food are increasing owing to climate change [9], while the economic burden of vibriosis affecting aquatic organisms (e.g., fish, bivalves, and crustaceans) farmed in aquaculture is estimated to be in the hundreds of billions of dollars worldwide [10].

### Eighteenth to nineteenth century

The first description of the genus *Vibrio* was made by Otto Frederik Müller in 1773 in his book *Vermium terrestrium et fluviatilium, seu Animalium Infusoriorum, Helminthicorum et Testaceorum, non marinorum, succincta historia* [45]. Later, in 1786 he expanded the description in his treatise on microscopic organisms that live in freshwater and marine habitats: *Animalcula Infusoria; Fluvia Tilia et Marina* [46]. Müller included 15 species in this genus, providing names and descriptions for each. However, almost all the species originally placed in this genus were later reclassified. For instance, species such as *Vibrio rugula, Vibrio undula, Vibrio serpens*, and *Vibrio spirillum* were reclassified by later researchers and placed in the genus *Spirillum* [11]. In 1835, the German scientist Christian Gottfried Ehrenberg described a hay/grass‑associated bacterium, *Vibrio subtilis*, later recognized as *Bacillus subtilis* [47]. In 1841, Fuchs described *Vibrio cyanogenus* and *Vibrio xanthogenus*, which were later identified as *Pseudomonas syncyanea* and *Pseudomonas synxantha*, respectively. Many of the bacterial species initially included in the genus *Vibrio* before 1854 have now been reclassified into other genera [48]. Interestingly, *Vibrio* is present in the first three‑kingdom tree of life published in 1866 by Ernst Haeckel in his *General Morphology of Organisms* [49], which is considered the earliest “tree of life” model of biodiversity. Indeed, Haeckel described *Vibrio* as “simple primordial cell masses, without a nucleus and without a shell or membrane, which remain at this lowest level of individuality throughout their lives” [49].

The first nonpathogenic *Vibrio* species isolated from the environment, namely *Vibrio fischeri* and *Vibrio splendidus*, were described by a Dutch microbiologist, Martinus Beijerinck, also known as the father of virology, in the late 1880s [50–52]. Luminous bacteria isolated by Beijerinck were sealed in glass ampoules in 1924 and 1925 and stored under the names *Photobacterium phosphoreum* and *Photobacterium splendidum* [53]. The Beijerinck strains are apparently the oldest bacterial cultures to be revived from storage and were later identified to form an evolutionarily distinct clade of *Vibrio* [53].

In 1893, Giovanni Canestrini reported epizootics in eels [54] (*Anguilla vulgaris*) associated with a bacterium initially described as *Bacillus anguillarum* and later reclassified as *Vibrio anguillarum*. This bacterium has been historically recognized as a major pathogen affecting marine animals [55]. An example is a disease observed in young Pacific salmon held in seawater ponds [56]. The disease, attributed to *V. anguillarum*, caused extensive hemorrhage in the muscles and internal organs of the fish and typically occurred from April or May, when temperatures increased, and persisted throughout the summer. Because the bacterial populations cultured from near‑shore waters and those associated with fish and shellfish were predominantly *Vibrio*, this genus attracted earlier attention from environmental microbiologists [1].

### Twentieth and twenty‑first centuries

At the end of the nineteenth century, the science of marine microbiology emerged as a relatively new discipline from the fusion of microbiology, oceanography, and marine biology, but it was only after the publishing of the foundational text *Marine Microbiology: A Monograph on Hydrobacteriology* in 1946 by Claude ZoBell [57] that bacteria began to be systematically isolated from the sea and characterized. The ease with which most *Vibrio* species are grown in the laboratory using Zobell (e.g., marine agar and broth) and other microbiological media paved the way for discovering new *Vibrio* from the environment.

*Vibrio harveyi* is a halophilic *Vibrio* species first described as *Achromobacter harveyi* by Johnson and Shunk in 1936, who named it in honor of Edmund Newton Harvey, a pioneer in the study of bioluminescence. It has been classified in *Lucibacterium* and *Beneckea* genera but is now included in the *Vibrio* genus. Many strains are bioluminescent, but also non‑luminescent strains have been described within the species. *V. harveyi* has been isolated from various geographical locations and from coastal and open ocean seawater, as well as from the surfaces of fish and squids [58]. Numerous papers have been published on its physiology and metabolism, and it is often used in bioluminescence studies as a model [5]. Mariners, on many occasions over the centuries, have reported observing “milky seas,” a mysterious phenomenon where the ocean shines intensely and uniformly at night. This light emission appears to be due to luminous *V. harveyi* strains living in association with colonies of the microalga *Phaeocystis*. However, the details of the formation mechanisms, spatial extent, global distribution, temporal variability, and ecological implications of milky seas remain almost entirely unknown [59].

On 20 and 21 October 1950, an outbreak of food poisoning due to the consumption of “*shirasu*” (whitebait) occurred in the cities of Osaka, Kishiwada, Kaizaka, and Izumi‑Sano in Japan [60]. During the outbreak, 272 individuals developed symptoms of acute gastroenteritis and 20 died. Initially, authorities suspected the outbreak to be a criminal case of poisoning. However, subsequent research led by Tsunesaburo Fujino at the Research Institute for Microbial Diseases (RIMD) of Osaka University succeeded in isolating *Pasteurella parahaemolytica*, from *shirasu* samples and post‑mortem material [60]. A few years later, in 1955, Iwao Takikawa isolated a similar bacterium from a food poisoning case that he named *Pseudomonas enteritis*. In the following years, the pathogenic microorganisms isolated were identified as belonging to the same species and reclassified as *Vibrio parahaemolyticus* [60, 61].

In 1965, Zen‑Yoji and collaborators [62] suggested that many summer outbreaks of food poisoning in Tokyo were caused by the consumption of sushi contaminated with *V. parahaemolyticus* biotype 1. In 1969, the “Kanagawa phenomenon” was described as the hemolysis of Wagatsuma blood agar by *V. parahaemolyticus* linked to its enteropathogenicity [63], and the year after, thermostable direct hemolysin (TDH) was identified as responsible for Kanagawa hemolysis.

Subsequently, it was discovered that *V. parahaemolyticus* had an annual cycle of abundance in marine waters and estuaries depending on temperatures and living in association with zooplankton [35, 64]. After the emergence of the pandemic O3:K6 strain (sequence type 3) in 1995–96 [65], this pathogen became one of the most globally significant marine pathogenic bacteria. Since then, it has been responsible for epidemics on many other continents [66, 67]. Nowadays*, V. parahaemolyticus* is the main cause of acute gastroenteritis in humans, caused by the consumption of raw, undercooked, or improperly processed seafood. *V. parahaemolyticus* pandemic ST3 and ST36 strains together with *V. cholerae* O1 are the only known examples of marine bacterial pathogens capable of transcontinental expansion [68]. In 1968, Sakazaki renamed *V*. *parahaemolyticus* biotype 2 to *V*. *alginolyticus* which can be pathogenic to both humans and marine animals [69].

*Vibrio natriegens* was first described as *Pseudomonas natriegens* by Payne in 1961, who isolated it from salt marsh mud on Sapelo Island off the coast of Georgia, USA [70]. *V. natriegens* utilizes a wider variety of carbon sources than other marine vibrios, making it easy to isolate and identify. It also has the shortest generation time (9.8 min) of any other bacterium [71], making it a popular species for use in scientific educational exercises and physiological studies [11]. Since then, *V. natriegens* has been isolated from coastal seawater in several locations [5]. *V. natriegens* has been considered a valid, faster‑growing alternative to *E. coli* to utilize as a model organism for molecular biology [72].

*Vibrio campbellii* is a halophilic species first described by Baumann in 1971, who isolated 60 strains from ocean water off the coast of Hawaii [73]. They noted considerable variation in the phenotypic properties of this large group but included all of them in *V. campbellii*. Grimes et al., in 1986, isolated 20 strains belonging to this species from pelagic water from Barbados to Puerto Rico to Bermuda, and concluded that *V. campbellii* is also found in the open waters of the Atlantic Ocean [74]. This finding established that *Vibrio* bacteria do not only inhabit coastal waters but also have an oceanic lifestyle [11].

*Vibrio vulnificus* was first isolated by the US Centers for Disease Control and Prevention (CDC) in 1964, although at that time it was misidentified as a virulent strain of *V. parahaemolyticus*. It was later recognized as a distinct species in 1976 thanks to the work of Hollis and collaborators [75] in the USA, while infection associated with this bacteria was first described as a necrotizing skin disease of unknown cause in Korea in 1979. Interestingly, infection cases possibly associated with this species were described in antiquity in the records of Hippocrates [76] and in the book *The Principle of Surgery* [77], published in 1801. Unlike other *Vibrio* species, *V. vulnificus* caused extraintestinal infections in humans and showed biochemical characteristics that distinguished it from other species. Initially, *V. vulnificus* was identified as a lactose positive (L+) *Vibrio*, and in 1979, it was observed that L+ *Vibrio* could cause infection, particularly during the summer [78]. *V. vulnificus* infections are relatively rare but extremely dangerous, with a high mortality rate of about 50%. It can cause necrotizing fasciitis, commonly known as flesh‑eating bacteria syndrome, a rapid and severe infection that destroys skin, fat, and muscle tissue. It can also lead to primary septicemia, a life‑threatening infection of the bloodstream, especially in individuals with compromised immune systems or underlying chronic conditions such as liver disease. In late 1979, Farmer proposed the name *V*. *vulnificus* (“vulni” = wound, “ficus” = to make) for this distinct pathogen owing to its association with a specific type of skin lesion [79].

*Vibrio mimicus*, first observed around 1980, emerged as a distinct species owing to unusual biochemical traits in *V. cholerae* strains. These distinctive traits were labeled as *V. cholerae*—lysine‑decarboxylase negative, *V. cholerae*—mannitol negative, or *V. cholerae*—sucrose negative. Through DNA hybridization and phenotypic analysis, researchers found that five out of the six unique biogroups were closely related to *V. cholerae* [80]. These were correctly identified as atypical strains of *V. cholerae*. However, a group of sucrose‑negative strains displayed significant divergence and distinct phenotypic characteristics compared with *V. cholerae*, leading the researchers to propose a new species named *V*. *mimicus* [80]. The name ‘mimicus’ refers to the fact that these strains mimic *V. cholerae* [80].

In 1984, Grimes and colleagues studied two urease‑positive halophilic *Vibrio* strains isolated from a brown shark (*Carcharhinus plumbeus*) that had died in captivity in a large aquarium [81]. One strain was identified as *Vibrio damsela*, a *Vibrio* species known to cause human wound infections and found in the marine environment [11], causing skin lesions on certain marine fish [82], first described by Love et al. in 1981. The other strain could not initially be identified. After further phenotypic testing and DNA–DNA hybridization, they concluded that this was a new species and named it *Vibrio carchariae*. Subsequent to the original report, *V. carchariae* has also been isolated from other sharks but has also been shown to be present in human clinical specimens. In 1989, Pavia et al. described a case where this species was isolated from a wound after a shark bite [83]. An 11‑year‑old girl was attacked by a shark on the coast of South Carolina. She suffered several deep lacerations to her left calf, which became infected after subsequent surgery. A culture of the infected wound yielded an unusual *Vibrio* that was later identified as *V. carchariae*. However, later taxonomic evidence showed that *V. carchariae* was a junior synonym of *V. harveyi* [84].

In January 1888, Martinus Beijerinck received a piece of salt pork that glowed in the dark from Mr. Enklaar of Deventer. Beijerinck’s laboratory notebook entry from January 12 notes that the flesh of the pork produced light and that some areas were brighter than others, observing a mixture of bacteria. Despite efforts to isolate the light‑producing species from the pork, his attempts were unsuccessful, possibly owing to the absence of sodium chloride in his medium [11], which consisted of pork, gelatine, peptone, and sodium carbonate. Nevertheless, his curiosity persisted, and on January 16, Beijerinck placed a piece of plaice fish on an open plate in his cellar. By January 22, the fish was glowing, enabling him to isolate light‑producing bacteria, such as *V. fischeri*, using a medium based on fish and seawater [50]. However, one of the processes behind bacterial bioluminescence, namely the global regulatory mechanism of quorum sensing, which is now known to control a diverse array of physiological activities in bacteria [11], was not yet understood at that time. Prior to 1994, quorum sensing was commonly referred to as “autoinduction” [7, 85]. Autoinduction was originally described for *V. fischeri* in the early 1970s. The experiments conducted by Kempner and Hanson in 1968 revealed the induction of bioluminescence in freshly inoculated *V. fischeri* [86]. The culture emitted luminescence in response to medium previously conditioned with the same strain. Nealson and collaborator in 1970 were the first to propose that autoinduction of luminescence in *V. fischeri* occurs at the transcriptional level and that the process is regulated by extracellularly secreted components [7, 87]. The term “quorum sensing” was introduced by Steven Winans in 1994, who wrote one of the first reviews on autoinduction in bacteria [88]. Furthermore, one of the most incredible and elegant forms of interaction and coevolution of *Vibrio* with aquatic organisms is the association between the Hawaiian bobtail squid, *Euprymna scolopes*, and the bioluminescent *Vibrio* bacterium that colonizes the light organ of the squid [89]. This was not the first discovery of a symbiotic relationship between *V. fischeri* and marine animals [90], but it is considered the most iconic one and has been studied for over 30 years as a model system for the colonization of animal epithelia by symbiotic bacteria [91]. This complex symbiosis involves colonization, quorum‑sensing‑induced bioluminescence, diurnal rhythm, maintenance, and dispersal, highlighting the intriguing interaction between *V. fischeri* and the Hawaiian squid. *V. fischeri* bioluminescence is also used in the Microtox bioassay to detect toxic substances in different substrates, where exposure to a toxic substance causes disruption of the respiratory process of the bacteria, resulting in reduced light emission.

In 1996, Kushmaro demonstrated for the first time that coral bleaching of *Oculina patagonica* was caused by a bacterial infection due to *Vibrio* AK‑1, later identified as *Vibrio mediterranei* (equivalent: *Vibrio shilonii* strain AK1) [92]. In 2002, a novel temperature‑dependent pathogen causing bleaching and lysis of the coral *Pocillopora damicornis* was isolated in Zanzibar. Although the causative agents of most coral diseases remain unknown, this particular strain caused rapid destruction of coral tissue within 2 weeks at water temperatures above 26 °C. It was classified as a member of the *Vibrio* genus and received the species name “*coralliilyticus*” owing to its deadly activity against corals [93]. Around the same time, *Vibrio aestuarianus* (Anguillarum clade) [94] and *Vibrio tasmaniensis* LGP32 [95] were found to cause mass mortality of the Pacific oyster *Crassostera gigas* in France. Several putative agents have now been identified to play a role in the onset of coral and bivalve diseases, many of which belong to the *Vibrionaceae* family [96, 97].

The availability of new resources and the release of chemical substances into the oceans have likely influenced the selection pressures also on *Vibrio* populations, leading to the emergence of new lineages and adaptations. An example is *Vibrio cyclitrophicus*, which was isolated from creosote‑contaminated marine sediments in 2001 [98]. This strain demonstrated the ability to utilize various polycyclic aromatic hydrocarbons as carbon substrates, including naphthalene, 2‑methylnaphthalene, and phenanthrene [98], resulting in a potential candidate for bioremediation process [98].

*Vibrio diazotrophicus*, a nitrogen‑fixing *Vibrio*, has been isolated from diverse sources, such as the gastrointestinal tracts of sea urchins collected in Nova Scotia, Canada and the surfaces of reeds growing in a drainage ditch in Kent, England [99]. The expanding role of vibrios in the environment, particularly in nutrient cycling, has begun to be recognized [10].

Ultimately, new *Vibrio* species have also been isolated from remote and unusual environments [100]. Notably, *Vibrio antiquarius* [101], *Vibrio diabolicus* [102], *Vibrio profundi* [103], and *Vibrio bathopelagicus* [104] isolated from the deep sea have genomes that encode virulence genes, suggesting that such genes may serve a fundamental ecological role than solely causation of human and animal diseases [101]. These strains offered unique opportunities to investigate the root of pathogenicity in environmental bacteria [101].

To date, more than 150 species of *Vibrio* have been described, almost all of which can be cultured by standard microbiological methods, an advantage that has allowed microbiologists to significantly expand the knowledge on the biology and ecology of these bacteria.

## Conclusions

Bacteria belonging to the genus *Vibrio* are undoubtedly important inhabitants of riverine, estuarine, and marine aquatic environments [1, 105]. Research on this group of bacteria has, over the years, led to a deeper understanding of the biology and ecology of significant human and animal pathogens. It has also contributed to the discovery of fundamental biological processes in bacteria and has led to relevant applications in the biotechnological field.

Among the essential elements impacting *Vibrio* life, climate change profoundly affects and will continue to affect the ecology of all *Vibrio* species through several mechanisms. These include direct effects, such as providing more favorable living conditions, e.g., warmer water temperatures and lower salinity [106, 107], as well as indirect effects, such as extreme weather events like rainfall and submersion waves that may inundate wastewater treatment stations, along with the influx of nutrients, diluted pharmaceuticals, and potentially resistant strains from hospital discharges. In addition, evidence has shown a positive relationship between *Vibrio* occurrence and extreme weather events, such as heatwaves [11, 108–110]. Drinking water and wastewater treatment facilities are also threatened by cyclones, which can cause population displacement and create precarious living conditions during the reconstruction period, factors that contribute to the spread of epidemics.

Projections indicate that, by 2100, under the least favorable climate scenario, coastal areas suitable for *Vibrio* colonization could expand by 38,000 km, with an increase in seasonal suitability of approximately 1 month every 30 years [111]. This underscores the importance of understanding and monitoring *Vibrio* as a “microbial barometer” of climate change [106]. It also highlights the need for integrated approaches that combine microbiology, genomics, epidemiology, and climate science to address the emerging threat of *Vibrio*‑associated diseases [111].

Plastic pollution in oceans and rivers is another emerging phenomenon that has increased dramatically in recent years and plays a significant role in bacterial ecology [112]. Plastic particles provide a medium for bacterial growth and, additionally, facilitate the long‑distance transport of bacteria. However, the implications of plastic pollution for *Vibrio* life strategies are still poorly understood [113].

As research into *Vibrio* evolution continues, scientists are gaining a better understanding of the genetic and ecological factors that have shaped this diverse group of bacteria. Such knowledge is essential for developing strategies to combat *Vibrio*‑related diseases, mitigate their impact on marine ecosystems, and anticipate future challenges in a changing world. Despite being one of the first discovered bacteria, and after almost two centuries of research, many fundamental aspects of the biology and ecology of *Vibrio* still remain an enigma.
